# Accuracy of microbial community diversity estimated by closed- and open-reference OTUs

**DOI:** 10.7717/peerj.3889

**Published:** 2017-10-04

**Authors:** Robert C. Edgar

**Affiliations:** Sonoma, CA, United States of America

**Keywords:** OTU, Alpha diversity, Beta diversity, QIIME, Open-reference, Closed-reference

## Abstract

Next-generation sequencing of 16S ribosomal RNA is widely used to survey microbial communities. Sequences are typically assigned to Operational Taxonomic Units (OTUs). Closed- and open-reference OTU assignment matches reads to a reference database at 97% identity (closed), then clusters unmatched reads using a *de novo* method (open). Implementations of these methods in the QIIME package were tested on several mock community datasets with 20 strains using different sequencing technologies and primers. Richness (number of reported OTUs) was often greatly exaggerated, with hundreds or thousands of OTUs generated on Illumina datasets. Between-sample diversity was also found to be highly exaggerated in many cases, with weighted Jaccard distances between identical mock samples often close to one, indicating very low similarity. Non-overlapping hyper-variable regions in 70% of species were assigned to different OTUs. On mock communities with Illumina V4 reads, 56% to 88% of predicted genus names were false positives. Biological inferences obtained using these methods are therefore not reliable.

## Background

Next-generation sequencing has revolutionized the study of microbial communities in environments ranging from the human body (Cho & Blaser, 2012; Pflughoeft & Versalovic, 2012) to oceans (Moran, 2015) and soils (Hartmann et al., 2014). Data analysis in such studies typically assigns sequences to Operational Taxonomic Units (OTUs). OTU assignment methods that use a pre-defined reference database of known sequences are called *reference-based*, while *de novo* methods construct clusters using only sequences found in the reads. Representative *de novo* methods include PyroNoise (Quince et al., 2009), UPARSE (Edgar, 2013), DADA2 (Callahan et al., 2016) and UNOISE2 (Edgar, 2017d), which attempt to infer correct biological sequences from noisy reads, and agglomerative methods implemented by DOTUR (Schloss & Handelsman, 2005), mothur (Schloss et al., 2009) and ESPRIT-Tree (Cai & Sun, 2011) which assign reads to clusters without attempting to predict which sequences are correct.

Probably the best-known reference-based method is *closed-reference* clustering (Rideout et al., 2014) as implemented by the *pick_closed_reference_otus.py* script in the QIIME package (Caporaso et al., 2010) and here called *Qclosed*, for *QIIME closed-reference*. By default, the reference database used by *Qclosed* is a subset of the Greengenes 16S rRNA sequence database (DeSantis et al., 2006) clustered at 97% identity. For brevity, I shall refer to the full Greengenes database as *GG* and the subset as *GG97*. Each sequence in GG97 defines an OTU. *Qclosed* processes a query sequence (typically, a read) by searching GG97 and assigning it to a reference sequence with ≥97% identity. If no match ≥97% is found, the query sequence is designated a *fail*. GG97 OTU assignments by *Qclosed* are used as the input for downstream analyses such as PICRUSt (Langille et al., 2013) which predicts a community metagenome and metabolic pathways by consulting pre-computed attribute tables for all sequences in GG97. Sequences with <97% identity to GG97 are discarded by *Qclosed*. QIIME *open-reference* clustering (Rideout et al., 2014) starts with *Qclosed* then performs *de novo* clustering on the fails (i.e., the queries that failed to match GG97 with ≥97% identity). At the time of writing, using the *pick_open_reference_otus.py* script is ”the preferred strategy for OTU picking among the QIIME developers” (http://qiime.org/tutorials/otu_picking.html, accessed 25th April 2017). I shall refer to the recommended protocol per the QIIME tutorials as QIIME*.

Previously published validations of closed- and open-reference OTUs include (Westcott & Schloss, 2015; Rideout et al., 2014). In Westcott & Schloss (2015), the authors compared several OTU methods using the Matthews Correlation Coefficient as a quality metric and found that the QIIME methods had low quality by this measure. In Rideout et al. (2014), the authors constructed OTUs using several methods on samples which were obtained *in vivo* and therefore have diversities which are unknown *a priori*. Results from different OTU assignment methods were compared on the same input data and shown to be consistent according to measured values for the number of observed OTUs, the Phylogenetic Diversity (Faith, 1992) and weighted UniFrac (Lozupone et al., 2007). However, agreement between different methods is not sufficient to show that these metrics are biologically realistic because incorrect results may be reproducible, e.g., due to unfiltered experimental artifacts in the reads.

The goal of this study was to evaluate estimates of diversity obtained by closed- and open-reference clustering methods in QIIME v1.9 using synthetic (*mock*) communities with known in-sample (*alpha*) and between-sample (*beta*) diversities. To investigate the impact of errors on diversity estimates, I used the following data as input: (1) known sequences obtained from finished genomes for the strains in the community, testing an ideal scenario where there are no errors; (2) known sequences to which a low rate of simulated errors were added; and (3) reads of mock communities generated on Illumina and 454 platforms.

## Methods

### Mock communities

Mock1 is the community with 27 strains used to validate DADA2 (Callahan et al., 2016). Mock3 is the HMP mock community (Haas, Gevers & Earl, 2011) with 21 strains and Mock2 is Mock3 plus one additional strain (*Candida albicans*). Mock3 contains one pair of strains (*S. aureus* ATCC BAA-1718 and *S. epidermidis* ATCC 12228) which have >97% identity for all primer pairs considered in this work and are therefore expected to fall into the same OTU when 97% clustering is used. By design, the Mock1 community has several pairs of strains with >97% identity over the sequenced region (V4) and should therefore ideally yield fewer than 27 OTUs. Mock3 has Even and Staggered sample types. Even samples are designed to have abundances to yield equal numbers of 16S rRNA genes for each strain while Staggered samples have uneven abundances ranging over three orders of magnitude. The Mock2 and Mock3 datasets considered here contain reads for both Even and Staggered samples which were combined before generating OTUs. The Mock1 sample has an uneven abundance distribution.

### Sequencing reads

Reads used in this study are summarized in Table 1. Bok is mock reads from Bokulich et al. (2013) and Koz is mock reads from Kozich et al. (2013). Set names starting with Hmp are from the Human Microbiome Project (HMP) (HMP Consortium, 2012). The set name indicates the hyper-variable regions sequenced and the direction of sequencing, e.g., HmpV13A sequenced the V1–V3 region in the forward direction and HmpV96A sequenced the V6–V9 region on the reverse strand. An A or B is appended to distinguish different runs. The Mock2/3 community has been sequenced in several different studies using different primer sets, enabling comparison of reads of different tags and different sequencing technologies (Illumina and 454 pyrosequencing). Reads in the Koz dataset that were assigned to mock samples include thousands of species from gut and soil samples due to cross-talk Edgar (2017c), i.e., assignment of reads to the wrong sample. Koz reads enable testing of a scenario where low- and high-diversity samples are multiplexed into the same sequencing run.

**Table 1:** Mock datasets used in this study. SRA is the NCBI Short Read Archive accession.

Set	Primers	Sample	Strains	Species	Genera	Families	SRA	Platform	# reads
Extreme	V4F, V4R	Mock1	27	26	11	7	SRR2990088	Illumina	1,256,239
Bok	V4F, V4R	Mock2	22	22	19	19	–	Illumina	7,056,809
KozV34	V3F, V4R	Mock3	21	21	18	18	–	Illumina	651,731
KozV4	V4F, V4R	Mock3	21	21	18	18	–	Illumina	4,758,584
KozV45	V4F, V5R	Mock3	21	21	18	18	–	Illumina	2,175,664
HmpV13A	V1F, V3R	Mock3	21	21	18	18	SRR053857	454	23,164
HmpV13B	V1F, V3R	Mock3	21	21	18	18	SRR053821	454	52,712
HmpV31A	V3F, V3R	Mock3	21	21	18	18	SRR053859	454	2,744
HmpV31B	V3F, V1R	Mock3	21	21	18	18	SRR053823	454	43,024
HmpV35A	V3F, V5R	Mock3	21	21	18	18	SRR053858	454	16,223
HmpV53A	V5F, V3R	Mock3	21	21	18	18	SRR053860	454	56,439
HmpV53B	V5F, V3R	Mock3	21	21	18	18	SRR053824	454	14,150
HmpV69A	V6F, V9R	Mock3	21	21	18	18	SRR053861	454	17,494
HmpV69B	V6F, V9R	Mock3	21	21	18	18	SRR053825	454	48,141
HmpV96A	V9F, V6R	Mock3	21	21	18	18	SRR053820	454	27,473
HmpV96B	V9F, V6R	Mock3	21	21	18	18	SRR053856	454	12,619

### Known tag sequences

I use the term *tag* to refer to the segment of the 16S rRNA gene between a given pair of primers. A tag is conventionally named by the hyper-variable region(s) it contains. For example, V4 is currently a popular tag for Illumina sequencing and V35 (i.e., V3–V5) was a popular tag for pyrosequencing. All strains in the Mock1, Mock2 and Mock3 communities have high-quality finished genomes, and the 16S rRNA sequences for these strains are therefore known. A given strain may have multiple small-subunit ribosomal RNA operons (paralogs) containing distinct 16S rRNA sequences. I constructed a reference database of known tags as follows. I used the SEARCH_16S algorithm (Edgar, 2017a) to search the genome of each strain and identify its full-length 16S rRNA sequence(s), as described in (Edgar, 2017b). For each pair of primers, I extracted the segment between the primer-matching loci. Up to two primer mismatches were allowed, ensuring that a tag was extracted for every strain from all full-length 16S rRNA sequences for every primer pair. For a given mock community and primer pair, the known tags are the sequences that would be obtained from the reads if there were no errors due to PCR and sequencing, or from noisy reads by a perfect denoiser. Using known tags as input to an OTU assignment method thus gives a lower bound on the number of spurious OTUs that could be achieved by minimizing or eliminating sequence errors. Each sequence was provided in two copies to avoid discarded singletons by QIIME*.

### Quality filtering

Per the QIIME tutorials for Illumina and 454 (http://nbviewer.jupyter.org/github/biocore/qiime/blob/1.9.1/examples/ipynb/illumina_overview_tutorial.ipynb and http://qiime.org/tutorials/tutorial.html respectively, accessed 25th April 2017), the recommended method for quality filtering is to use the *split_libraries_fastq.py* script with default parameters. QIIME v1.9 does not support stand-alone quality filtering to the best of my knowledge, and I therefore implemented the Bokulich et al. Phred (*Q*) score filtering method in my own Python script (provided in Supplemental Information 2).

### Simulated sequencing error

To investigate the effects of sequencing error, I generated every possible sequence variant with a single substitution (*1-sub.*) of the known tag sequences. With the popular V4 tag (∼250 nt), one substitution per sequence models a base call error rate of ∼1/250 = 0.004 (equivalent to a Phred score of Q24), which is a low error rate (high quality) by current standards; on the longer V35 and V69 tags it is ∼1/500 = 0.002 (Q27). There are 250 × 3 = 750 possible 1-sub. variants of a given V4 tag sequence, and with the deep sequencing achieved by Illumina, most or all possible 1-sub. variants of more abundant strains may be found in the reads due to sequencing errors, polymerase substitution errors and chimeras (Edgar, 2016a). A V4 sequence with one substitution is 249∕250 = 99.6% identical to the correct sequence; two such sequences are 248∕250 = 99.2% identical to each other. This variation is comparable to typical intra-strain variation due to paralogs, and is small compared to typical intra-species variation due to differences between strains. All 1-sub. variants for a given strain would therefore ideally be assigned to the same OTU.

### Beta diversity

I calculated beta diversity using weighted UniFrac (Lozupone et al., 2007) and the weighted Jaccard distance (Jaccard, 1912). UniFrac considers OTUs to be similar if they are close to each other in a tree. Weighting uses OTU frequencies rather than presence-absence so that low-abundance OTUs contribute less to the measure and the metric is less sensitive to sampling effects. For a pair of samples *X* and *Y*, the weighted Jaccard distance (*J*) is calculated as:

\begin{eqnarray*}J=1- \left\{ {\sum }_{i}\min ({x}_{i},{y}_{i}) \right\} \left/ \right. \left\{ {\sum }_{i}\max ({x}_{i},{y}_{i}) \right\} . \end{eqnarray*}

Here, *x*_*i*_ is the frequency of OTU *i* in sample *X* and *y*_*i*_ is the frequency of OTU *i* in sample *Y*. The frequency is *n*_*i*_∕*N* where *n*_*i*_ is the number of reads assigned to OTU *i* in the sample and *N* is the total number of reads for the sample. If *J* is 1, then no OTU is present in both samples (equivalently, every OTU is found in only one of the samples), indicating that the samples are maximally different. If the frequencies are identical in both samples then *J* = 0, so when comparing two replicate samples, *J* would ideally be zero. To correct for differing numbers of reads per sample, a random subset of 5,000 reads was extracted from each sample before calculating distances.

### Beta diversities from closed-reference OTU tables

OTU tables were generated by *Qclosed* for all mock samples containing the Mock2 or Mock3 community. These were considered to contain the same community for this analysis (in fact they contain 21 identical strains while Mock2 has one additional species). I calculated the weighted Jaccard and weighted UniFrac metrics for every pair of samples using the *Qclosed* OTU tables. Histograms were created to show the distribution of these beta diversities by binning metric values into intervals of 0.05.

### Non-overlapping tags

It has been claimed (Rideout et al., 2014; Caporaso et al., 2010) that closed-reference clustering enables comparison of non-overlapping tags, and the QIIME documentation states “You **must** use closed-reference OTU picking if you are comparing non-overlapping amplicons, such as the V2 and the V4 regions of the 16S rRNA” (http://qiime.org/tutorials/otu_picking.html accessed 25 April 2017, emphasis in original). Presumably, this claim is based on the assumption that non-overlapping tags for a given strain will usually be assigned to the same OTU by closed-reference. To investigate this, I identified the subset (*GG-tagsX*) of GG-tags which has binding loci for all primer pairs. The GG-tagsX subset comprises only 60,470 sequences (4.8% of the full GG database) because most Greengenes sequences are truncated such that they lack binding sequences for the V1 forward primer and V9 reverse primer (Edgar, 2017b). I assigned all tags in GG-tagsX to OTUs by *Qclosed* and calculated the probability that a given pair of tags in the same sequence would be assigned to the same OTU.

### Taxonomy prediction accuracy

I measured the accuracy of QIIME* taxonomy predictions on the mock samples as follows. Predictions were assessed by considering the set of genus names in the designed community to be complete and correct for each sample. In fact, it is possible that some strains might be missing from the reads, and unexpected strains might be present due to contaminants and cross-talk; results should be interpreted accordingly. For each sample, I calculated the following values: *N*, the number of distinct correct names; *M*, the number of distinct predicted names; *TP*, the number of true positives (distinct predicted names which are correct); *FP*, the number of false positives (distinct predicted names which are not correct), and *FN*, the number of false negatives (correct names which were not predicted). Typical classification accuracy metrics such as sensitivity, specificity, precision and recall apply to binary classifiers for which true negatives should be considered. Here, the classification is not binary, and true negatives cannot occur under the operational assumption that all genera in the sample are known. I therefore used the following metrics: *discovery rate* (*DR*), the fraction of correct genus names which are predicted, i.e., *DR* = *TP*∕*N*; *true prediction rate* (*TPR*), the fraction of predicted genera which are correct, i.e., *TPR* = *TP*∕*M*; and *false prediction rate* (*FPR*), the fraction of predicted genera which are incorrect, i.e., *FPR* = *FP*∕*M*.

### Alpha diversity and rarefaction analysis by QIIME

To validate alpha diversity analysis using the recommended QIIME scripts, I used the Bok reads. These contain two Even and two Staggered samples of the Mock2 community, which has 22 strains. Rarefaction curves were generated by QIIME using the procedure described in the Illumina tutorial: *split_libraries_fastq.py* with forward reads only, *pick_open_reference_otus.py* and *core_diversity_analyses.py*. Default parameters were used for all these scripts, as in the tutorial, except for sampling depth which is left for the user to decide. I tried a range of depths from one thousand to one million.

### Chimera identification

I used the UCHIME2 algorithm (Edgar, 2016a) to identify chimeric sequences. To obtain a conservative estimate I used high-confidence mode, which sets parameters designed to minimize false positives at the expense of allowing more false negatives. The number of chimeras found by this method is therefore likely to be an underestimate.

### Sensitivity of database search

*Qclosed* uses the USEARCH algorithm as implemented by uclustq v1.2.22 (Edgar, 2010) to search GG97. USEARCH is a heuristic algorithm designed to optimize speed at the possible expense of sensitivity. To investigate the cause of failures to match GG97, I used GG-tags as input to *Qclosed*. All GG-tags sequences are present in GG, and a failure to match GG97 could therefore be due to a false negative by uclustq, or because a tag is <97% even though its full-length sequence is ≥97%. These cases were distinguished by measuring the identity of all sequences in GG-tags with GG97 according to uclustq.

## Results

### Numbers of OTUs on mock reads

Table 2 summarizes the total number of OTUs reported by each method on the mock reads (Table 1). On the Illumina datasets, QIIME* reported 4,482 OTUs on Bok, 298 on Extreme and 1,607, 2,857 and 5,824 respectively on KozV34, KozV4 and KozV45. Richness as measured by the number of OTUs is thus greatly inflated compared to the number of strains or species in the mock samples. On all datasets except Extreme, many of the OTUs were predicted to be chimeric. The absence of detectable chimeras in the Extreme reads is expected because the strains were amplified separately (some intra-strain chimeras may be present, but these would have very low divergences and would therefore not be detected by UCHIME2 in high-confidence mode).

**Table 2:** Mock OTUs reported by *Qclosed* and QIIME*. The first two columns give the numbers of OTUs reported by QIIME closed-reference (*Qclosed*) and the recommended QIIME protocol (QIIME*). The second two columns show the numbers of chimeras in the OTU sequences for Qclosed and QIIME* respectively as predicted by the high-confidence mode of UCHIME2.

Set	Strains	*Qclosed* OTUs	QIIME* OTUs	*Qclosed* chimeras	QIIME* chimeras
Bok	22	955	4,482	41	703
Extreme	27	343	298	0	0
KozV34	21	531	1,607	39	899
KozV4	21	2,263	2,857	47	816
KozV45	21	1,312	5,824	61	2,983
HmpV13A	21	30	565	13	220
HmpV13B	21	36	1,414	11	456
HmpV31A	21	56	536	14	284
HmpV31B	21	60	1,171	20	584
HmpV35A	21	127	679	20	128
HmpV53A	21	218	2,143	37	575
HmpV53B	21	138	739	23	223
HmpV69A	21	61	973	33	387
HmpV69B	21	75	2,562	56	728
HmpV96A	21	68	1,606	11	539
HmpV96B	21	59	792	9	304

### OTUs assigned to known tags

Generating OTUs from the known sequences in the mock communities is an idealized scenario where there is no sequence error in the input data. Results are shown in Table 3 (first two columns). The richness values reported by *Qclosed* are close to the number of mock strains, though this is somewhat misleading because some strains are absent due to fails and in other cases a strain is *split*, i.e., it has distinct paralog sequences which are assigned to two or more different OTUs (Table 4). Failed strains are reflected in the increase in richness by QIIME* over *Qclosed*. Fails would naively not be expected in this test because all mock strains are present in GG. An order of magnitude or more increase in richness is seen when substitutions are added to simulate a low rate of errors (0.004 base call error rate, or Q24) due to PCR and sequencing (Table 3, last two columns).

**Table 3:** Richness of OTUs assigned to known tag sequences in Mock3. Here, richness is the number of OTUs reported by closed-reference (*Qclosed*) and the recommended QIIME protocol (QIIME*), respectively. In the first two columns, input is the known tag sequences for the strains in the Mock3 community, modeling an idealized case where all biological sequences in the sample are correctly identified, e.g., by a perfect denoiser. QIIME* richness is given as the number of additional OTUs found compared to *Qclosed*. Naively, we would expect *Qclosed* to assign all tags to OTUs because they all belong to strains found in GG. In the last two columns, the 1-sub. variants of each tag sequence are included, i.e., all possible sequences that differ by a single substitution, modeling a very low rate (0.2 to 0.4%) of incorrect bases due to PCR and sequencing.

Tags	*Qclosed*	QIIME*	*Qclosed* + 1 sub.	QIIME* + 1 sub.
Mock3-V13	24	+2	217	+4
Mock3-V34	16	+4	327	+14
Mock3-V35	17	+4	306	+14
Mock3-V4	21	+0	450	+11
Mock3-V45	22	+1	442	+16
Mock3-V69	21	+0	190	+8

**Table 4:** *Qclosed* OTU assignments for known tags in Mock3. The table shows OTU identifiers assigned by *Qclosed* for tags in the known 16S rRNA genes in the HMP mock community. Ideally, a given species would always be assigned to the same OTU regardless of which tag or which paralog is being classified, but this is true only of *D. radiodurans*. Shading indicates cases where two or more tags were assigned to the same OTU; singletons are underlined.

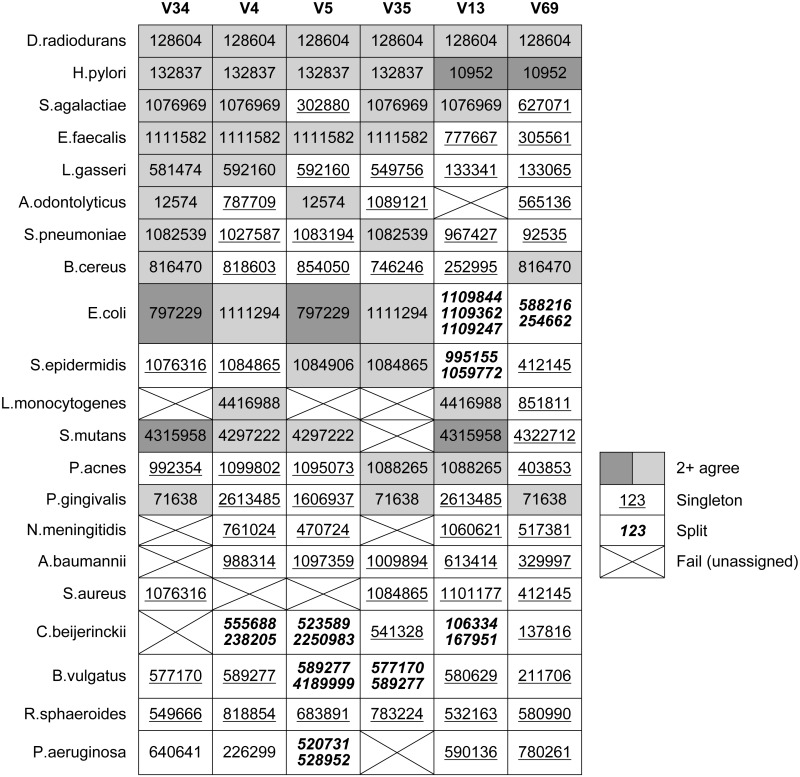

**Table 5:** Probability that different tags in a given 16S rRNA sequence are assigned to the same OTU by *Qclosed*. For each pair of tag sequences in GG-tagsX, the table shows the fraction which were assigned to the same OTU by the QIIME closed-reference method. Pairs which overlap have darker shading.

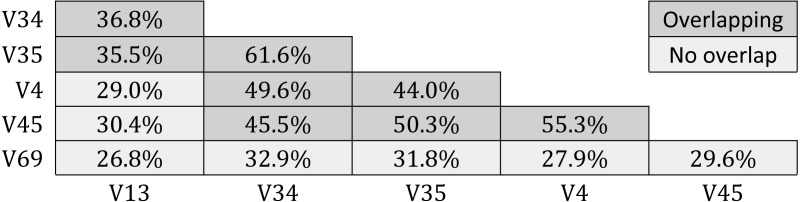

Table 4 shows which OTUs were assigned to the known tags in each Mock3 strain by *Qclosed* (correct sequences only, without substitutions). This is an idealized test in two respects: there is no sequence error, and all full-length sequences in the sample are present in the original reference database (GG). Despite this, at least one strain fails to be assigned to an OTU for all tags except V69. With V35, four strains are lost, representing 20% of the community: *A. odontolyticus*, *L. monocytogenes*, *N. meningitidis* and *P. aeruginosa*. With the currently popular V4 tag, *S. aureus* is lost. Some strains are split over two or three OTUs, with *E. coli* split over three OTUs in the case of V13. Splitting is promiscuous when simulated sequence error is introduced (Table 3).

### Non-overlapping tags

Table 4 shows OTU assignments by *Qclosed* for known tags in the mock strains. This shows that different tags from a given strain are usually assigned to different OTUs. Most OTU assignments are singletons; i.e., different from all other tags for the strain. The same OTU is assigned to all tags for only one species, *D. radiodurans*.

Results for GG-tagsX are shown in Table 5. Overlapping pairs were assigned to the same OTU with probabilities ranging from 35.5% (V13 and V35) to 61.6% (V35 and V34). Probabilities tend to increase with increasing overlap, as might be expected. Pairs with no overlap are assigned to the same OTU with low probabilities ranging from 26.8% (V13 and V69) to 32.9% (V34 and V69). All five tags were assigned to the same OTU for only 10,954/60,470 (18.1%) of the full-length sequences used to construct GG-tagsX.

### Quality filtering

The QIIME quality filtering algorithm has previously been shown to allow many reads with >3% errors which can cause large numbers of spurious OTUs (Edgar & Flyvbjerg, 2014). I found that this filter rejected only 0.3% of the reads in the Koz dataset. This is because only *Q* scores ≤3 (error probability > 0.5) are considered as potentially unacceptable, and such scores are rare in the Koz FASTQ files. The filter has more effect on the Bok reads, rejecting 7.1% of the reads. This is because it rejects runs of bases with *Q* = 2 which are commonly found at the 3′  ends of these reads. All reads in the Extreme dataset were passed by the filter, suggesting that the original reads may have been quality-filtered before they were deposited in the SRA.

### Alpha diversity analysis and rarefaction analysis by QIIME

**Figure 1:**
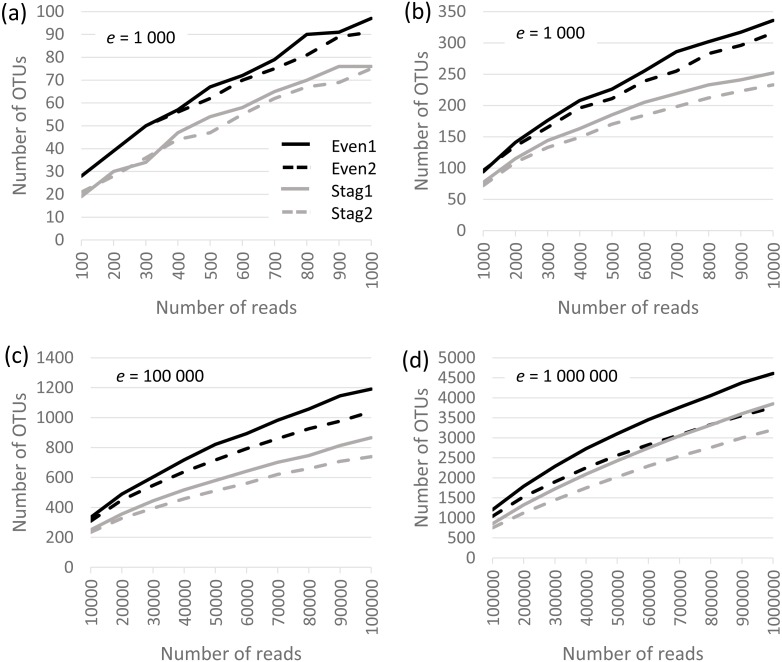
Rarefaction curves for Bok reads generated by QIIME. There are two Even and two Staggered samples of Mock3 (22 strains). The *e* parameter is the number of reads per sample.

Analysis was performed on the Bok reads, which have 22 strains in the mock community. Results are shown in Fig. 1, which show that richness is inflated from a factor of ∼5× (∼100 OTUs at a depth of one thousand reads per sample) to ∼200× (∼5,000 OTUs at a depth of one million). No convergence is observed in the rarefaction curves, reflecting that almost all OTUs are due to errors which accumulate at a roughly constant rate as the number of reads increases. Thus, at all tested read depths, the reported diversity mostly reflects uncorrected experimental artifacts rather than biologically meaningful groups.

### Beta diversity of closed-reference OTUs

**Figure 2:**
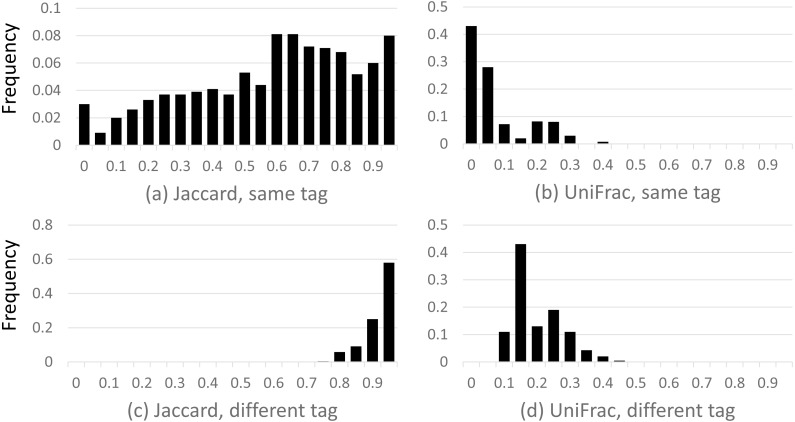
Distribution of closed-reference beta diversities for all pairs of Mock2/3 samples. The histograms show the distribution of weighted Jaccard (A, C) and weighted UniFrac (B, D) distances on all pairs of samples containing Mock2 or Mock3. A zero value for the Jaccard or UniFrac distance indicates maximum similarity between a pair of samples; one indicates maximum difference. Histograms (A) and (B) show the distribution when the same tag is sequenced (e.g., V4), histograms (C) and (D) when different tags are sequenced (e.g., V13 and V69). The *y* axis is the frequency, calculated as (number of sample pairs having distances which fall into a given bin) divided by (total number of sample pairs).

Results are shown in Fig. 2, which shows a dramatic difference between the Jaccard and UniFrac distances. Most Jaccard distances are large, incorrectly indicating low similarity between the samples, especially when different tags are compared. This is readily explained by the ubiquitous and inconsistent splitting of species into different OTUs by *Qclosed*. By contrast, most UniFrac distances are small, correctly suggesting high similarity between the samples. Thus, UniFrac is more tolerant of species splitting by the closed-reference approach.

### Accuracy of taxonomy prediction

Results are shown in Table 6. The genus discovery rate (DR) ranged from 50% (HmpV13A) to 88% (two Illumina and three 454 datasets). This is consistent with the sensitivity of 77% for V4 sequences of known genera previously measured for the QIIME default taxonomy prediction method (Edgar, 2016b). The genus false prediction rate (FPR) was >50% for all Illumina datasets while FPR was much lower on the 454 reads, ranging from 5% (HmpV53B) to 11% (HmpV35A and HmpV53A). A high error rate is expected on the Koz datasets because many reads assigned to mock samples contain valid sequences from other samples due to cross-talk (Edgar, 2017c). However, the reason for the high error rates on Bok and Extreme compared to the 454 datasets is not clear. Naively, it might be expected that the FPR would correlate with the number of OTUs because each spurious OTU gives a new opportunity for a prediction error, but the number of OTUs is lower on Extreme (298) than any of the 454 datasets (minimum 536 on HmpV31A).

**Table 6:** Taxonomy assignment accuracy for QIIME* OTUs.

Platform	Set	*N*	*M*	*TP*	*FP*	*FN*	*DR*	*TPR*	*FPR*
Illumina	Bok	19	39	16	23	3	84%	41%	58%
	Extreme	11	16	7	9	4	63%	43%	56%
	Koz.V34	18	37	16	21	2	88%	43%	56%
	Koz.V4	18	134	16	118	2	88%	11%	88%
	Koz.V45	18	73	17	56	1	94%	23%	76%
454	HmpV13A	18	10	9	1	9	50%	90%	10%
	HmpV13B	18	11	10	1	8	55%	90%	9%
	HmpV31A	18	12	11	1	7	61%	91%	8%
	HmpV31B	18	10	9	1	9	50%	90%	10%
	HmpV35A	18	18	16	2	2	88%	88%	11%
	HmpV53A	18	18	16	2	2	88%	88%	11%
	HmpV53B	18	17	16	1	2	88%	94%	5%
	HmpV69A	18	17	15	2	3	83%	88%	11%
	HmpV69B	18	16	15	1	3	83%	93%	6%
	HmpV96A	18	14	13	1	5	72%	92%	7%
	HmpV96B	18	14	13	1	5	72%	92%	7%

### Errors in the Greengenes taxonomy hierarchy

In QIIME v1.9, taxonomy annotations for the GG97 OTUs are specified in the file *97_otu_taxonomy.txt* (https://github.com/biocore/qiime-default-reference/blob/master/qiime_default_reference/gg_13_8_otus/taxonomy/97_otu_taxonomy.txt.gz, accessed 25th April 2017). In these annotations, 36 genera are placed in two or more families, violating the structure required for a valid taxonomy. To give some examples, genus *Rhodospirillum* is placed in family *Rhodospirillaceae* (e.g., in the annotation for OTU 326714), which is correct according to Bergey’s Manual (Bergey, 2001), and also in family *Alcaligenaceae* (OTU 119663). Genus *Vibrio* is in *Vibrionaceae* (OTU 9303, correct), and *Pseudoalteromonadaceae* (OTU 1115975). Genus *Flexibacter* is placed in three families: *Cytophagaceae* (OTU 1142767, correct), *Flammeovirgaceae* (OTU 4447268), and *Flavobacteriaceae* (OTU 1136639).

### Sensitivity of GG97 clustering and database search

I found 41 pairs of sequences in GG97 with 100% identity, for example 4365807 and 4374946 (see Supplemental Information 2 for complete list). These are errors by the method used to construct GG97, strongly suggesting that there are many more pairs with >97% identity, though these cannot be unambiguously identified because different methods do not always agree on the identity of a given pair of sequences, and the method used to create GG97 is not documented to the best of my knowledge. *Qclosed* results on the GG-tags dataset are summarized in Table 7. From 0.6% (V45) to 6.9% (V13) of tags failed (i.e., were not assigned to a GG97 OTU). Some of these failures are due to tags with <97% identity as shown in the last column in Table 7. The remainder is due to false negatives by the database search method. Notably, some of the fails are tags extracted from the GG97 subset, which therefore have 100% identity with at least one full-length sequence in GG97.

**Table 7:** *Qclosed* results for GG-tags. Columns are Sequences, the number of tag sequences (and as a fraction GG sequences these represent due to a truncated full-length sequence or >2 primer mismatches); GG97 tags, the number of GG97 sequences from which this tag was extracted (and as percentage of all GG97 sequences); Fails, the total number of fails (and as a percentage of all tested tags); GG97 fails (and as a percentage of GG97 tags), and <97%, the number of tags with <97% identity with the full-length GG97 database (and as a fraction of GG-tags).

Tag	Sequences	GG97 tags	Fails	GG97 fails	<97%
V13	266,317 (21.1%)	46,426 (46.7%)	18,404 (6.9%)	186 (0.4%)	10,386 (3.9%)
V34	1,236,137 (97.9%)	93,280 (93.9%)	13,956 (1.1%)	180 (0.2%)	6,179 (0.5%)
V35	1,240,170 (98.3%)	94,370 (95.0%)	18,477 (1.5%)	880 (0.9%)	6,201 (0.5%)
V4	1,245,904 (98.7%)	93,610 (94.2%)	13,018 (1.0%)	152 (0.2%)	6,228 (0.5%)
V45	1,249,794 (99.0%)	94,621 (95.3%)	7,866 (0.6%)	33 (0.0%)	4,999 (0.4%)
V69	100,470 (8.0%)	13,848 (13.9%)	2,422 (2.4%)	25 (0.2%)	1,706 (1.7%)

## Discussion

### Alpha diversity estimates by QIIME are inflated

The default alpha diversity metrics reported by the QIIME *core_diversity_analysis.py* script are richness (number of OTUs), Chao-1 (Chao, 1984) and Phylogenetic Diversity (*PD*) (Faith, 1992). Richness was grossly inflated on the Illumina mock datasets. Chao-1 has a lower bound of richness, which is already inflated, so Chao-1 would also be over-estimated. Chao-1 values were not explicitly considered here because the QIIME calculation is incorrect: the number of singletons appears in the formula, but singletons are discarded by the recommended QIIME procedure (here called QIIME*). PD was designed to enable comparison of genetic and phenotypic diversity in different communities with the goal of prioritizing conservation efforts (Faith, 1992). It is calculated using a tree such that OTUs which are close in the tree contribute less to diversity. This is analogous to unweighted UniFrac, because tree distance is considered but abundance is not. UniFrac is relatively robust against spurious OTUs (at least, on mock samples, when weighted to suppress low-frequency OTUs), and is it therefore possible that PD could also be robust. I did not attempt to validate PD in this work because it is not clear to me how to interpret its numerical value on a single sample, and in particular how to determine whether an estimated value on a mock sample is biologically realistic.

### Weighted UniFrac is tolerant of spurious OTUs

Weighted UniFrac was found to report small distances between identical (or very similar) mock samples despite high rates of spurious OTUs and substantial divergences in which spurious OTUs were present (as shown by the large distances according to the weighted Jaccard metric). This is presumably explained because UniFrac considers OTUs to be similar if they are close in the tree, and spurious OTUs due to sequence errors tend to be close to the correct OTU. However, if UniFrac is not sensitive to such errors, then it is necessarily also insensitive to genuine biological differences which induce similar differences between the OTUs in a pair of samples; e.g., the replacement of a species in one sample by a closely related species in the other. Beta diversity metrics such as Jaccard which do not consider tree distance are less tolerant of spurious OTUs but more sensitive to variations in closely related OTUs.

### Failures to assign known sequences cannot be avoided by closed-reference

Table 7 shows that a known sequence (i.e., a sequence which is present in GG) may fail to be assigned to an OTU for two reasons: (1) a false negative by the database search, and (2) a tag has identity <97% despite having ≥97% identity over the full-length sequence. False negatives could be addressed by an improved database search method, but some tags have lower identities than their full-length sequences, and failures of type (2) are therefore unavoidable in a closed-reference method. With open-reference, failures of both types (1) and (2) cause *de novo* OTUs to contain known sequences and these OTUs could be regarded as unexpected or erroneous. Some sequences which are very close to GG should fail according to the design of the algorithm. For example, consider a sequence *S* in GG which has exactly 97% identity with the most similar GG97 sequence (*R*), and a sequence *T* which is not in GG and has one substitution compared to *S*. It is very likely that *T* is <97% with GG97 (because 97% of possible substitutions are at positions where *S* and *R* are identical, and substitutions at those positions necessarily reduce identity between *T* and *R*), in which case *T* would be a correct fail [*sic*] by the design of the algorithm despite being almost identical to a GG sequence.

### Inflated diversities are primarily caused by inadequate error filtering

*Qclosed* and QIIME* gave biologically reasonable numbers of OTUs with error-free sequences but inflated numbers with a low rate of simulated errors, which strongly suggests that many, probably most, of the spurious OTUs obtained with noisy reads are caused by inadequate error filtering. This is consistent with an earlier study which observed that a large number of reads with >3% errors and high diversity are allowed by the QIIME quality filter (Edgar & Flyvbjerg, 2014). Spurious OTUs are also caused by chimeras, which are known to be ubiquitous in 16S rRNA amplicon sequences (Haas, Gevers & Earl, 2011) but are not filtered by *Qclosed* or QIIME*. This issue could be mitigated but not fully solved by adding a chimera filtration step, noting that the best current algorithms cannot reliably detect chimeras in reads that are quality-filtered but not denoised (Edgar, 2016a).

### Inflated diversities on mock tests suggest that similar results may occur in practice

Mock communities have low diversity, which raises the question of whether comparable results should be expected on communities with higher diversities. While there is insufficient evidence to support a robust claim, I believe that the number of spurious OTUs obtained on mock samples is probably representative of numbers obtained in practice. I will briefly summarize the argument here; more details are given in Supplemental Information 1. Spurious OTUs are primarily caused by chimeras, splitting due to paralogs, and substitution, insertion and deletion (SID) errors due to PCR and sequencing. Chimeras form preferentially between sequences with higher identity, and samples with low taxonomic diversity are known to occur in practice (Ravel et al., 2011). Such samples are likely to have higher chimera rates than the mock communities considered here. Splitting due to paralogs will tend to increase with richness because each strain adds a new opportunity to split. Rates of PCR and sequencing error should average out over different template sequences to a reasonable approximation, and the overall SID error rate should therefore not strongly depend on diversity. Then, if a sample with higher diversity is divided into subsets (call them *mock-like*) of 20 template sequences, the reads of each mock-like subset will induce a similar number of spurious OTUs to the same number of reads of a mock community. Combining the mock-like subsets then indicates that there will be a comparable number of spurious OTUs overall.

##  Supplemental Information

10.7717/peerj.3889/supp-1 Supplemental Information 1 Supplementary Note

10.7717/peerj.3889/supp-2 Supplemental Information 2 Supplementary files (data and scripts, .tar.gz format) Data and scripts to facilitate reproducing results.

## Additional Information and Declarations

### Data Availability

The following information was supplied regarding data availability: Data and scripts to facilitate reproducing results have been uploaded as Supplemental Files.
