# Supplementary material for: Accuracy of microbial community diversity estimated by closed- and open-reference OTUs
Source: PeerJ. 2017 Oct 4;5:e3889. doi: 10.7717/peerj.3889 (PMC5631090; doi:10.7717/peerj.3889)
Supplement: Supplemental Information 1 [file peerj-05-3889-s001.pdf]

Supplementary Note

Accuracy of microbial community diversity estimated by closed- and open-reference OTUs

Robert C. Edgar

The number of spurious OTUs probably does not strongly depend on diversity

While there is insufficient evidence to support a robust claim, I believe it is plausible that the number of spurious OTUs generated by a given method does not strongly depend on community structure, with some exceptions. Known reasons for spurious OTUs include contaminants, cross-talk, paralog splitting, chimeras, polymerase copying errors (which can cause substitutions, insertions and deletions in a read sequence compared to the correct sequence) and sequencer errors (also substitutions, insertions and deletions). Contaminants and cross-talk are presumably not influenced by community structure.

Splitting due to paralogs

Presumably, a single strain should be assigned to a single OTU. If a given strain has paralogs with differing sequences, and these sequences are assigned to more than one OTU (as seen for some strains in Table 4 in the main text), then richness will be inflated. The excess number of OTUs due to paralog splitting will tend to increase in samples with higher diversity because adding a new strain to the sample may cause a new split but is unlikely to cause a previously-split strain to merge back into a single OTU.

Chimeras

Sequences with high identity are more likely to form chimeras (Haas *et al.*, 2011). The chimera formation rate should therefore correlate with the probability that a randomly

chosen pair of template sequences has high identity. The mock communities considered here contain from eleven (Mock1) to 19 genera (Mock2) and from seven (Mock1) to 19 families (Mock2), as shown in Table 1 in the main text. A random pair of mock community templates will therefore usually belong to different families, and an *in vivo* community with few dominant genera could have comparable or higher average sequence similarity. For example, the human vaginal microbiome is often dominated by members of the *Lactobaccilus* genus (Ravel *et al.*, 2011) and it is therefore likely that a vaginal sample would have a higher chimera formation rate than a mock sample because a random pair of amplicons will often be derived from the same genus. Even if the chimera frequency is low, other sources of error can result in high diversity of erroneous sequences. This is illustrated by the Mock1 reads which have few or no inter-strain chimeras because the strains were amplified separately, but nevertheless gave large numbers of spurious OTUs with both *Qclosed* and *QIIME** (Table 2 in main text).

Substitution, insertion and deletion (SID) errors due to PCR and sequencing.

Call a read *harmful* if it has >3% SID errors. With a 97% threshold, harmful reads cause spurious OTUs. Reads with <3% errors can also cause spurious OTUs; I will neglect this scenario for simplicity because similar arguments apply. I will also neglect the possibility that a read with >3% errors might be the only read for a given strain, in which case its OTU might be considered valid for some purposes, e.g. calculating diversity. Such reads can be neglected to a reasonable approximation because they are surely rare even in samples with high diversity, as shown by the following reasoning. Let h be the frequency of harmful reads. Most reads are correct or have <3% errors so h is small. Let K be the number of singleton strains, i.e. strains having exactly one read. The number of singleton strains with

a harmful read will then be approximately hK, i.e. a small fraction of K. Thus, even if singleton strains are common, those with harmful reads will nevertheless be rare.

With the simplifications and caveats described above, a read falls into a spurious OTU if, and only if, it is harmful, i.e. has >3% SID errors. Therefore a new harmful read will necessarily cause a new spurious OTU unless it falls into an existing spurious OTU, i.e. is sufficiently similar to a previously-generated harmful read.

Frequencies for SID errors, and hence the rate of harmful reads due to SID errors, depend on several known factors including the sequencing platform (e.g., 454 or Illumina), the sequence of the template (e.g., the lengths of its homopolymers), and the PCR protocol (e.g., the chosen polymerase and number of cycles). I would therefore expect the frequency of harmful reads from a given template to be primarily determined by its sequence, with biases that depend on the experimental protocol (PCR and sequencing platform). The experimental protocol and number of reads is assumed to be the same for all samples, and while the fraction of reads with >3% no doubt varies somewhat between samples, the variation is probably not very large because biases will tend to average out, and there is no reason to believe mock samples have unusual biases. Therefore I would expect that:

> To a reasonable approximation (a) the fraction of harmful reads (i.e., with >3% SID errors) is independent of the sample composition, and (b) mock samples have rates of harmful reads comparable to rates in samples encountered in practice. (1)

Two harmful reads that fall into the same spurious OTU must be generated from the same template sequence (or two very similar template sequences), and have similar errors. In a sample with high diversity, each new harmful read is therefore likely to be a novel error, i.e. one that is not close enough to a previous harmful read to fall into the same spurious OTU. A novel harmful read creates a new spurious singleton OTU, and by (1) it would then follow that the number of spurious OTUs will be comparable for mock samples and samples encountered in practice. If the diversity is lower, especially if there are a few highly abundant templates, then there are more opportunities for errors to be reproduced, which will reduce the total number of spurious OTUs caused by a given number of harmful reads. Thus, the number of spurious OTUs due to SID errors may in fact tend to be lower in samples with low diversity, such as a mock community. This conclusion assumes that singleton OTUs are retained, as with the *Qclosed* method.

If singleton OTUs are discarded, as with *QIIME**, then forming a spurious OTU requires that an error is reproduced well enough that two harmful reads fall into the same OTU, and the rate of forming spurious OTUs will be more dependent on biases. Now imagine dividing a high-diversity sample into mock-like subsets of, say, 20 strains. If the number of spurious OTUs for a mock sample increases approximately linearly with the number of reads, as seen for *QIIME** in Fig. 1 in the main text, and the mock-like subsets of a high-diversity sample have comparable biases to a mock sample, then the total number of spurious OTUs will be approximately the same as a single mock community with the same total number of reads (proof below). The linear relationship probably does not hold with very low read depths per template sequence, which give too few opportunities for two similar harmful

reads to occur. Therefore, if most reads in a sample are derived from templates with very low depth, then the number of spurious OTUs may be lower than a mock sample after singleton OTUs have been discarded.

Proof that summing over mock-like subsets gives the same number of spurious OTUs when there is a linear dependence on the number of reads

Let the number of reads be n and b be the number of spurious OTUs caused by those reads. If the dependence is linear, then there is a constant r such that $b = r\,n$. For a mock sample, let N be the total number of reads, and B_{mock} be the total number of spurious OTUs. Then $B_{mock} = r\,N$. Now consider a high-diversity sample H and divide it up into mock-like subsets, each with 20 strains. Let the jth subset have n_j reads. Assuming that a subset of H with 20 strains and n_j reads produces the same number of spurious OTUs as a mock sample with n_j reads, then each subset will produce $b_j = r\,n_j$ spurious OTUs and the total number of spurious OTUs is $B_H = \sum_j b_j = \sum_j r\,n_j = r \sum_j n_j$. By assumption, the samples have the same number of reads so $\sum_j n_j = N$ and hence $B_H = r\,N = B_{mock}$.

References

Haas B, Gevers D, Earl A. (2011). Chimeric 16S rRNA sequence formation and detection in Sanger and 454-pyrosequenced PCR amplicons. *Genome Res* 494–504.

Luo C, Tsementzi D, Kyrpides N, Read T, Konstantinidis KT. (2012). Direct comparisons of Illumina vs. Roche 454 sequencing technologies on the same microbial community DNA sample. *PLoS One* **7**. e-pub ahead of print, doi: 10.1371/journal.pone.0030087.

Ravel J, Gajer P, Abdo Z, Schneider GM, Koenig SSK, McCulle SL, *et al.* (2011). Vaginal microbiome of reproductive-age women. *Proc Natl Acad Sci U S A* **108 Suppl**: 4680–7.
